# Dysregulation of T Lymphocyte Proliferative Responses in Autoimmunity

**DOI:** 10.1371/journal.pone.0106347

**Published:** 2014-08-29

**Authors:** Sydney K. Elizer, Andrew F. Marshall, Daniel J. Moore

**Affiliations:** 1 Department of Pediatrics, Ian Burr Division of Endocrinology and Diabetes, Vanderbilt University, School of Medicine, Nashville, Tennessee, United States of America; 2 Department of Pathology, Microbiology, and Immunology, Vanderbilt University, School of Medicine, Nashville, Tennessee, United States of America; INSERM-Université Paris-Sud, France

## Abstract

T cells are critically dependent on cellular proliferation in order to carry out their effector functions. Autoimmune strains are commonly thought to have uncontrolled T cell proliferation; however, in the murine model of autoimmune diabetes, hypo-proliferation of T cells leading to defective AICD was previously uncovered. We now determine whether lupus prone murine strains are similarly hyporesponsive. Upon extensive characterization of T lymphocyte activation, we have observed a common feature of CD4 T cell activation shared among three autoimmune strains–NOD, MRL, and NZBxNZW F1s. When stimulated with a polyclonal mitogen, CD4 T cells demonstrate arrested cell division and diminished dose responsiveness as compared to the non-autoimmune strain C57BL/6, a phenotype we further traced to a reliance on B cell mediated costimulation, which underscores the success of B cell directed immune therapies in preventing T cell mediated tissue injury. In turn, the diminished proliferative capacity of these CD4 T cells lead to a decreased, but activation appropriate, susceptibility to activation induced cell death. A similar decrement in stimulation response was observed in the CD8 compartment of NOD mice; NOD CD8 T cells were distinguished from lupus prone strains by a diminished dose-responsiveness to anti-CD3 mediated stimulation. This distinction may explain the differential pathogenetic pathways activated in diabetes and lupus prone murine strains.

## Introduction

The development of autoimmune disorders is under constant investigation from clinical and basic immunologic perspectives. Among these areas of research, investigation into autoimmunity in the NOD mouse has been significant in elucidating the ontogeny of autoimmune disease. However, it is not always clear how revelations gained in this system can be applied in other models of autoimmunity. Nonetheless, the NOD mouse can be induced to develop autoimmunity towards a number of target tissues other than the islets of Langerhans. While the selective loss of islet beta cells is well characterized, this murine strain is also highly susceptible to the induction of a panoply of autoimmune syndromes. Notably, the NOD mouse can develop hemolytic anemia, thyroiditis, encephalomyelitis, sialitis, and a lupus-like disorder [1], [2], [3], [4], [5], [6], [7], [8], [9], [10]. This pattern of disease susceptibility suggests the existence of overlapping immunologic perturbations among the NOD and other murine models of autoimmunity. Determining the mechanistic underpinning of these findings may further our understanding of common derangements that predispose to autoimmunity.

Among regulatory elements of immune effector functions, the CD4 T lymphocyte compartment plays a predominant role in the initiation of the immune response. Since autoimmune diseases manifest diverse pathogenic mechanisms, a perturbation in immune regulation by CD4 T lymphocytes may be a common phenotype driving these disorders. In this regard, it has been amply demonstrated that the appropriate differentiation of CD4 T cells toward regulatory/effector mechanisms is intimately linked to a precise pattern of proliferation. A proscribed number of cell divisions is required for CD4 T cells to acquire the capacity to secrete particular cytokine profiles and to undergo activation induced cell death (AICD), a paramount mechanism in protection from autoimmunity [11], [12], [13], [14], [15].

We have previously reported that CD4 T lymphocytes in the NOD mouse exhibit an aberrant division profile highlighted by their inability to achieve advanced numbers of divisions following polyclonal activation [16], a finding in agreement with several other published observations [17], [18], [19]. As the NOD mouse demonstrates susceptibility to a lupus-like syndrome, we investigated whether the aberrancy in the activation profile of CD4 T lymphocytes might be shared with murine models of spontaneous lupus [9], [10], [20], [21], [22]. For this purpose, we utilized the well-characterized spontaneous murine models of this disease, the MRL and the NZBxNZW F1 (NZBW) strains, to ascertain the characteristics of CD4 T cell activation. We demonstrate that an aberrant CD4 T cell division profile is shared by all three autoimmune strains, a finding that implicates the quality of CD4 T cell activation as a crucial determinant of autoimmune disease progression. This phenotype is not due to an intrinsic defect in CD4 T cell function but can instead be attributed to the antigen presenting cell compartment. Deficiencies in professional APC function leave T cells reliant on B cells for costimulation, a finding that partially explains the B cell dependency of these diseases. We have also extended our analysis to the CD8 compartment where the NOD strain is distinguished by a unique activation profile that may explain the differential expression of spontaneous autoimmunity among the strains investigated here.

## Results

### A basic phenotype shared among autoimmune strains

**Figure 1:**
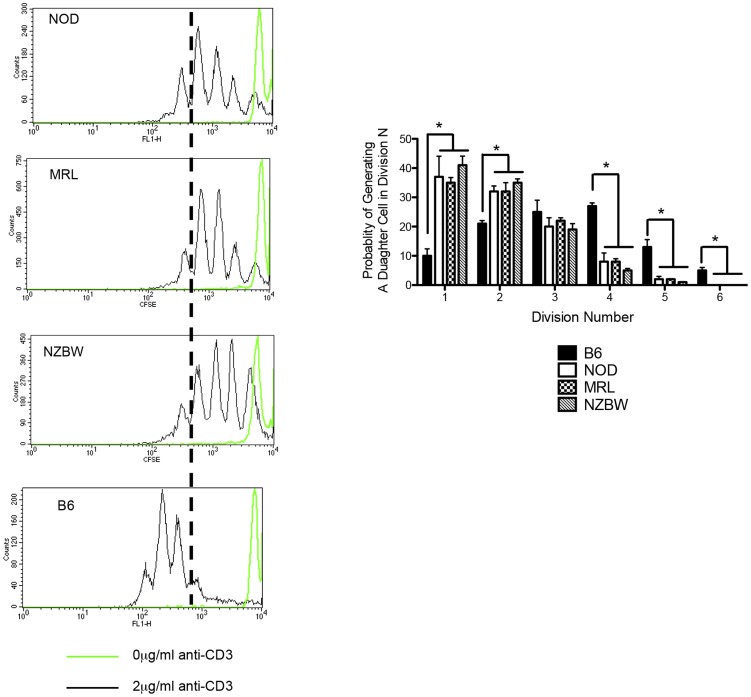
CD4 T cells isolated from autoimmune strains exhibit truncated cell division profiles. A) Splenocytes from NOD, MRL, NZBW, or C57BL/6 female mice at 8–11 weeks of age were labeled homogeneously with CFSE and incubated with 2 µg/ml anti-CD3 and 4 µg/ml anti-CD28 for 65 hours (dotted histogram); unstimulated cells are shown for comparison (solid line). The dotted line demarcates the third division in each graph. B) The probability of reaching any division is identical among autoimmune strains. The probability that a dividing precursor generates a daughter cell in division N was calculated under maximally stimulating conditions (2 µg/ml anti-CD3/28, 65 hours) and represented as a bar graph. An activated CD4 T cell from any autoimmune strain is unlikely to produce a daughter cell in divisions 4–6 (probability<10%) while there is a significant chance for this event in the normal strain B6 (probability>40%). The distribution of B6 daughter cells was significantly different from all other strains in each division except division 3 (*p<0.01, ANOVA followed by post-test, B6 was compared to each other strain, n>6). All data are representative of at least three separate experiments in each group.

We first determined whether the division aberrancy we had demonstrated among CD4 T lymphocytes in the NOD mouse could also be observed in the MRL and NZBW strains. Splenocytes were harvested from each autoimmune strain or from the non-autoimmune control C57BL/6 (B6) strain at 8 to 11 weeks of age and stimulated with anti-CD3 and anti-CD28 at a concentration known to induce maximal proliferation of both NOD and B6 CD4 T cells [16]. We have also studied the activation of splenocytes from the BALB/c strain as a second control group; as these activations were in all ways identical to the B6 strain and previously reported, the data in the present work is confined to the B6 strain [16]. Division history was followed with the vital fluorescent dye CFSE by flow cytometry after 65 hours of *in vitro* culture. CD4 T cells from all autoimmune strains exhibited identical proliferation profiles; they were able to generate few daughter cells beyond the third division (Fig. 1a). Specifically, while more than 60% of the daughter cells in B6 splenocyte cultures were found in divisions 4–6, less than 20% of progeny cells in the autoimmune cultures achieved such advanced division.

To facilitate comparison of the data from repetitions of the experimental protocol, we developed a mathematical process to analyze and display the proliferative data. As we are primarily interested in the behavior of the precursor cells that defines the range of specificities available following exponential expansion, we back-calculated the number of precursors that would be sufficient to compose each final division peak. We then represented the data as the probability that a given precursor (and hence specificity) would be present in a given division. This analysis allows us to predict the potential for any antigen-reactive cell to reach any division stage and hence better appreciate the frequency of avoidance of tolerogenic mechanisms that require the coordinated progression of an activated CD4 T cell through at least four rounds of cell division [14], [15], [23], [24].

Intriguingly, the three autoimmune strains demonstrated indistinguishable probabilities of generating a daughter cell in any division peak (Fig. 1b); moreover, these probabilities differ markedly from that seen for the non-autoimmune B6 strain. An activated precursor from each autoimmune strain has about a 10% chance of generating a daughter cell beyond the first 3 divisions. In contrast, an activated CD4 T cell from the B6 strain has an almost 40% chance of achieving this advanced level of cell division. This finding indicates that an activated autoantigen reactive cell in the non-autoimmune background would be nearly four times more likely to achieve the advanced cell divisions where cytokine deviation and AICD are known to occur.

### Dose Response Characteristics of Autoimmune CD4 T cells

**Figure 2:**
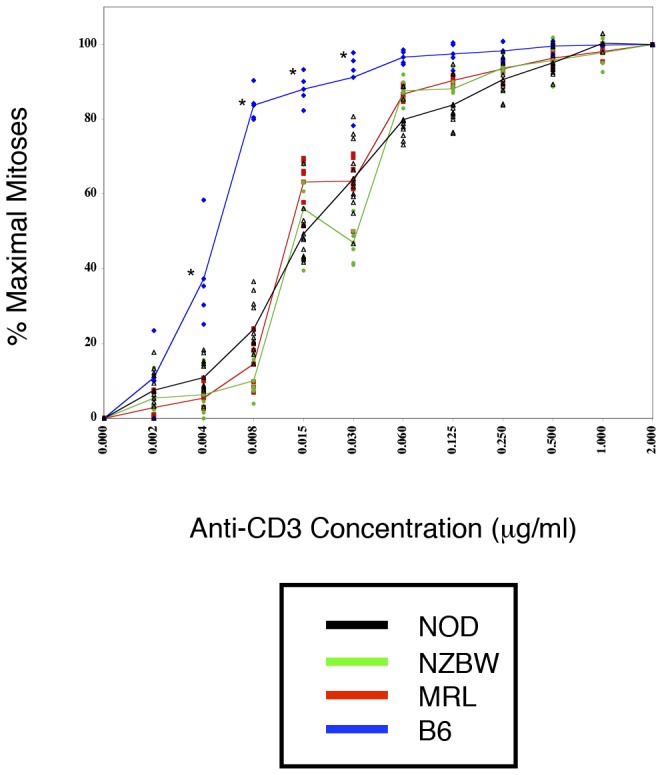
Dose responsiveness of autoimmune CD4 T cells to anti-CD3 mediated stimulation. The number of mitoses per 10,000 CD4 T cells was determined for cultures stimulated with a range (0–2 µg/ml) of anti-CD3 concentrations; each culture received 4 µg/ml anti-CD28. The percent of the maximal mitosis is shown plotted against the dose of anti-CD3. The autoimmune strain CD4 T cells exhibit dose-response curves that are less responsive than the normal control B6. Significant differences in response are noted at the lower concentrations of anti-CD3 (*p<0.01, B6 vs all other strains, ANOVA followed by Bonferroni post-test). It is important to note that 100% represents the maximal number of mitoses achieved by that strain; the maximum for the normal strain B6 is greater than that for the autoimmune strains. Data are from three separate experiments (n>6 for all strains).

Having demonstrated a profound diminishment in autoimmune strain CD4 T cell division at maximal concentrations of anti-CD3 antibody, it was also important to assess the reactivity of these cells to other doses of this stimulus. This analysis is particularly important as the levels at which autoantigens are presented *in vivo* remain unknown. In Figure 2, dose-response curves for each autoimmune strain are presented and compared to the normal B6 curve. As the number of CD4 T lymphocytes present in splenocyte preparations from each strain is highly strain specific, we performed calculations based on normalized cell numbers following flow cytometric analysis. This analysis permitted examination of the activation properties inherent in whole splenocyte cultures, which were likely to be most reflective of the internal milieu of these animals. For each strain, the data is reported as a percent of maximal mitosis. It is also important to note that these maxima differ among the strains; the maximum proliferation achieved by CD4 lymphocytes on the B6 background is markedly greater than that achieved by the autoimmune strains (Fig. 1a). Even at the highest doses of antibody, the proliferation of the autoimmune strains is much less than that in prototypical non-autoimmune strains.

The overlap among the dose-response curves for the autoimmune strains is apparent, as is their marked deviation from the normal B6 curve (Fig. 2). Extrapolating from the curves, we note that while B6 CD4 T cells reach the 50% activity level at 0.005 µg/ml anti-CD3, the autoimmune strains reach the half-maximal level at 0.02 µg/ml. This four-fold difference in dose-responsiveness coupled with the truncated division profile indicates a profound diminishment in CD4 T cell activity in the autoimmune strains.

### Autoimmune strain CD4 T cells are intrinsically normal

The stimulation of whole splenocytes with soluble mitogenic antibody demands cooperation between T cells and APCs in order for cell activation and division to progress [25]. Therefore, the alterations we have noted in autoimmune strain CD4 T cell activation may be due either to an intrinsic defect in the CD4 T cell compartment or to aberrancies in the APC compartment with which these T cells must interact. To address this question, we employed a second assay utilizing plate-bound antibody, a stimulus that activates T cells in the absence of APCs.

**Figure 3:**
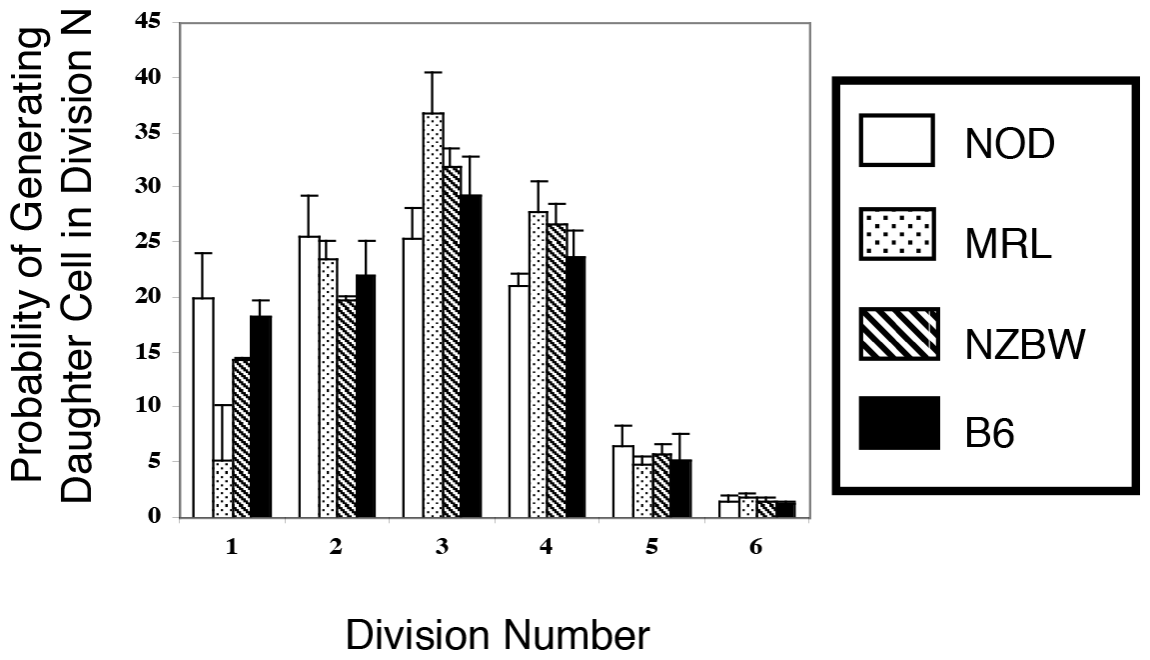
Plate bound stimulation normalizes the responsiveness of autoimmune CD4 T cells. Splenocytes were harvested from each strain, CFSE labeled, and stimulated in culture plates that had been previously coated with anti-CD3 and anti-CD28. The probability that a stimulated precursor will produce a daughter cell in any division is calculated as described in Figure 1. Following plate bound stimulation, this measure is shared among all strains. No significant differences were detected. Data are obtained from three independent experiments (n>6 for all strains).

Compared to stimulation with soluble anti-CD3 (Fig. 1a), CD4 T cell division following plate-bound stimulation resulted in a pronounced leftward shift of the autoimmune strain profile and a close similarity to the division profile exhibited by the B6 CD4 T cells. Using the probability distribution calculus described above, we demonstrate a normalization of division activity in these autoimmune strains (Fig. 3). Under plate-bound stimulation conditions, the autoimmune strains divide as well or better than their non-autoimmune B6 counterparts. This finding suggests that CD4 T cells from each autoimmune strain are intrinsically normal despite aberrancies in their activation under conditions of soluble antibody stimulation.

### Autoimmune strains rely on B cell mediated co-stimulation

The plate bound stimulation suggested a deficit within the antigen presenting cell compartment as the root cause of the hypoproliferative phenotype we have described. To further dissect this phenomenon, we attempted to discern whether we could identify a functional deficit in any APC subset. A number of studies have indicated that the autoimmune disease processes studied here rely on B lymphocytes for their development; we therefore sought to assess the responsiveness of splenocyte preparations that were devoid of B lymphocytes [16], [26], [27], [28], [29].

**Figure 4:**
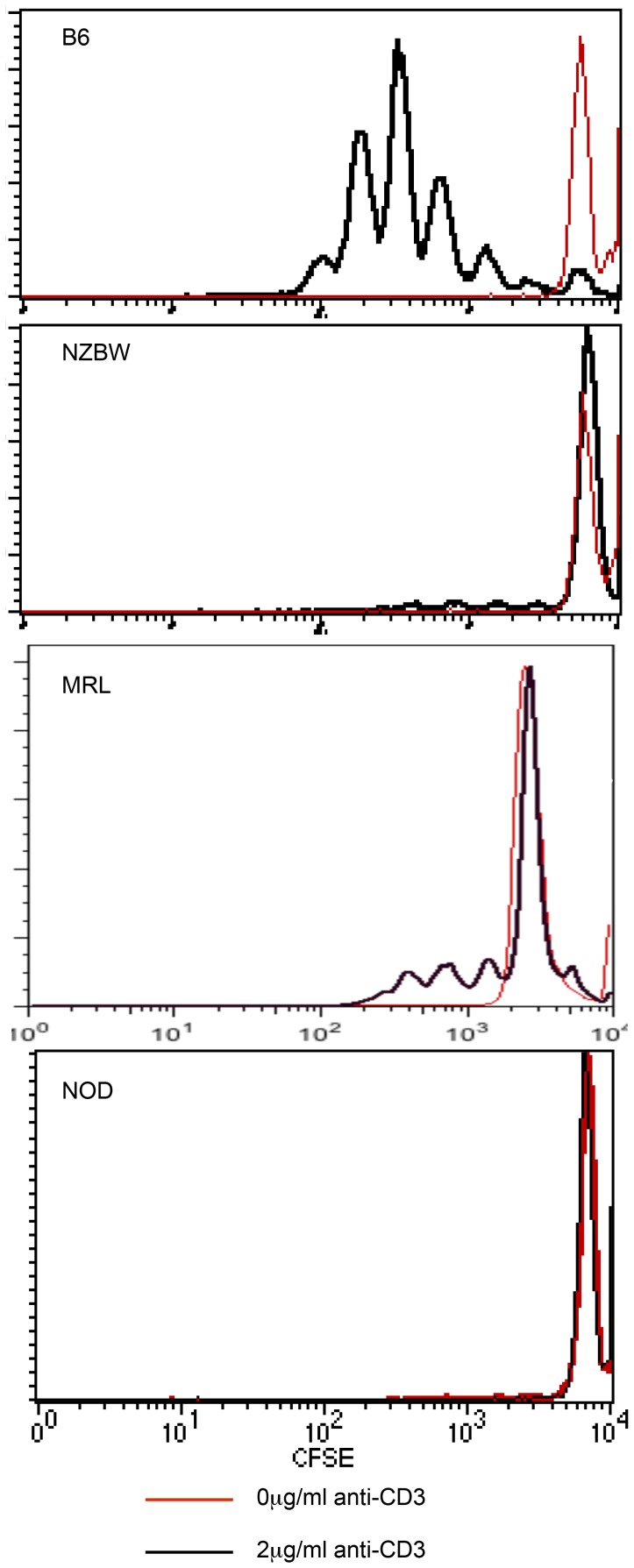
B lymphocytes are required for proliferation of T cells in autoimmune prone lineages. Splenocytes harvested from B6, NOD, MRL, or NZBW were depleted of B cells by MACS with anti-B220 antibodies. The resultant populations were stimulated with 2 µg/ml anti-CD3 and 2 µg/ml anti-CD28 for 65 hours (black histogram) or left unstimulated (red histogram). At that time, minimal proliferation was detected in B cell depleted cultures in autoimmune-prone mice whereas B6 splenocytes maintained normal proliferation in the absence of B cells. Data are representative of three to four separate experiments.

For this purpose, splenocytes were depleted of B cells using the MACS magnetic separation technique. B cells were selectively depleted with the use of anti-B220 antibody. Depletion was confirmed by flow cytometry with anti-CD19 antibody and was >95% efficient in all cases. The resultant B cell depleted fractions were CFSE-labeled and stimulated with soluble mitogenic antibody. While the control B6 strain retained the ability to proliferate robustly (Fig. 4), neither the NOD, MRL, nor the NZBWF1 strains were able to proliferate in the absence of this lymphocyte subset. This finding suggests that B cells are requisite co-activators of T cells in these autoimmune prone murine strains and that the remaining APC compartments (macrophages, dendritic cells) possess functional deficiencies that contribute to autoimmunity.

### Restricted cell division protects autoimmune strain CD4 T cells from activation-induced death

One potential consequence of restricted mitotic activity for CD4 T cells is the inability to achieve activation-induced cell death (AICD), a relevant mechanism for the clearance of autoreactive specificities [15], [30], [31]. On the other hand, it is possible that CD4 T cells from the autoimmune strains fail to reach advanced divisions because these cells possess enhanced susceptibility to AICD. To discriminate these alternatives, we determined the frequency of cell death in dividing CD4 T cells from these autoimmune strains.

**Figure 5:**
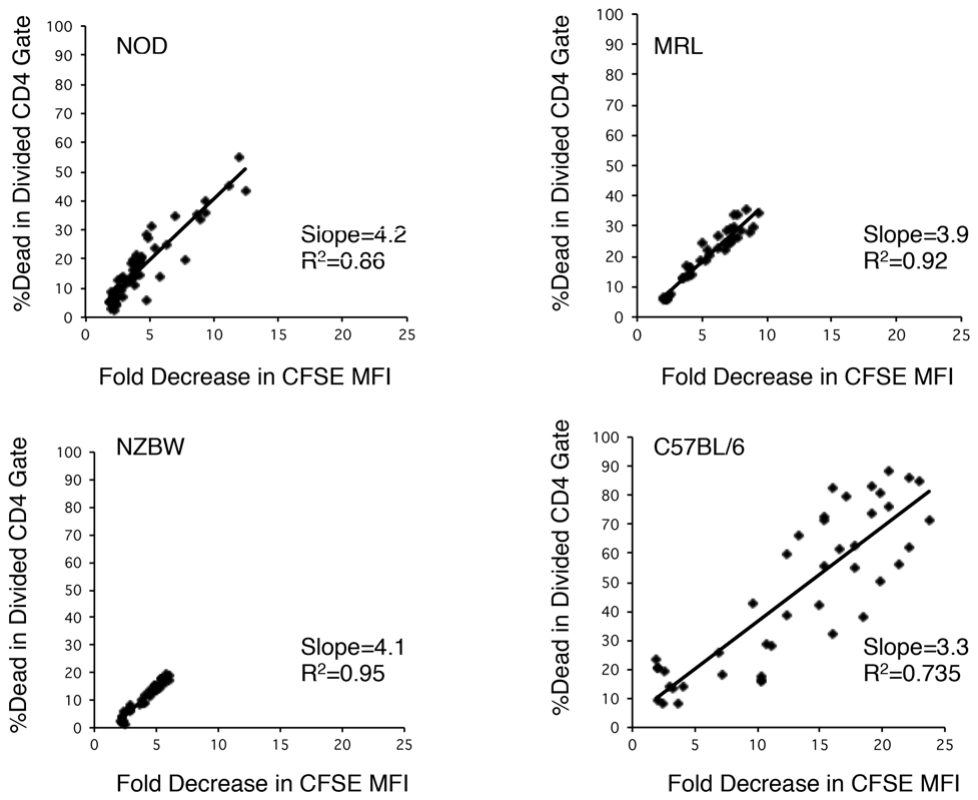
Susceptibility to activation induced cell death is governed by cell divisions. The percentage of cell death as detected by 7-AAD staining in the divided CD4 T cell gate is plotted against the fold reduction in median fluorescence intensity as an indicator of the extent of cell division. Dots from twelve stimulation conditions in four separate experiments are plotted. Stimulated cells from C57BL/6 animals achieve nearly twice the maximal division seen for the autoimmune strains as indicated by the maximum reduction in CFSE intensity and, therefore, they undergo more AICD. However, at shared points along the ordinate, all four strains demonstrate similar levels of cell death.

For all strains, whether autoimmune or normal, there exists a linear relationship between cell death and the extent of cell division (Fig. 5). In fact, the slope of each line is nearly equivalent in all cases. However, since the autoimmune CD4 T cells do not achieve the same extent of cell division, fewer cells undergo AICD. While maximally stimulated B6 CD4 T cells experienced a 25-fold reduction in fluorescence intensity of CFSE and a corresponding high degree of cell death, the autoimmune strains could not exceed a 12-fold reduction. Nonetheless, the autoimmune strain cells experienced an appropriate level of cell death, as compared to the normal B6 strain, for that amount of division. When higher levels of mitotic activity are induced as in the plate bound stimulation assays, the higher levels of cell death projected by the linear regression are observed (data not shown) further indicating that the actual process of AICD is not disrupted in the autoimmune strains.

### Distinct Activation Profile Exhibited in the CD8 Compartment of the NOD Mouse

**Figure 6:**
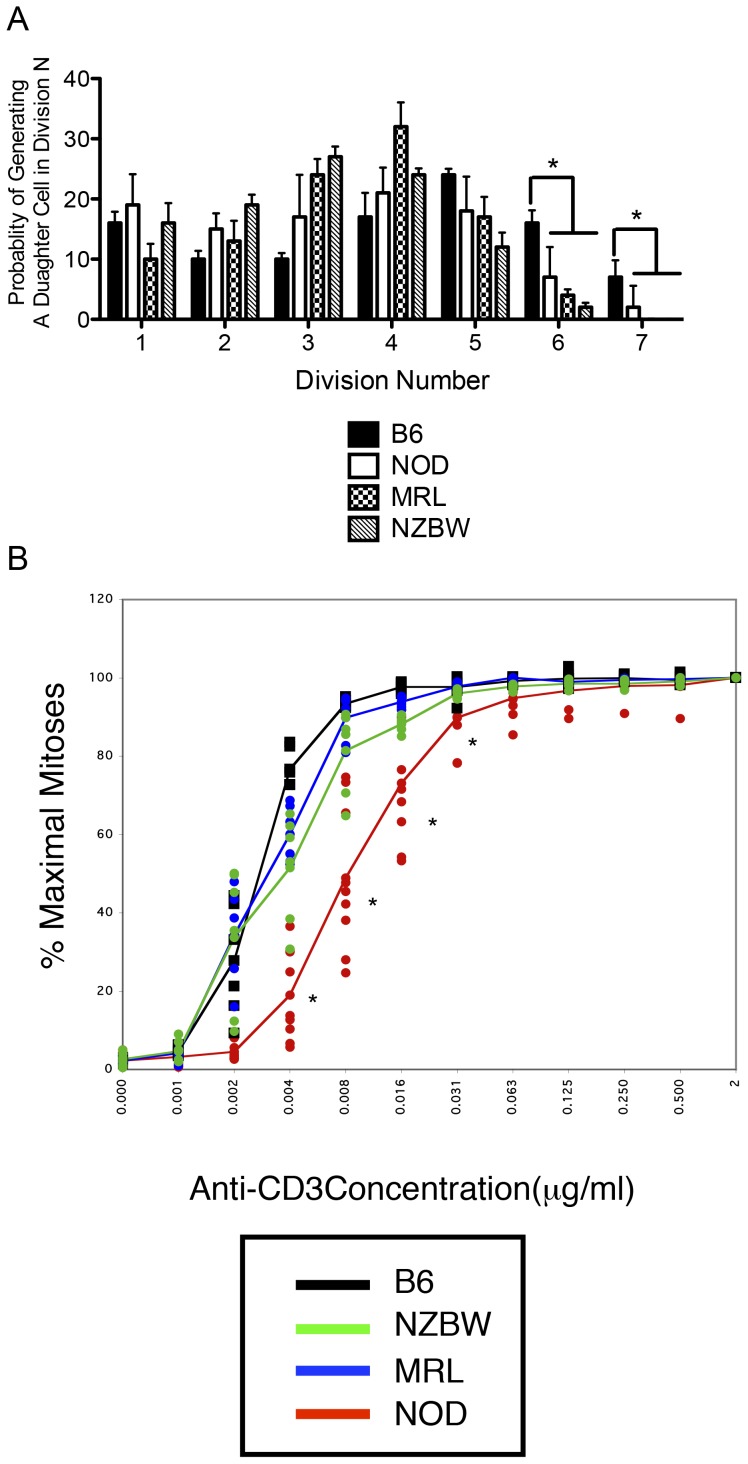
CD8 T cells from all strains are able to reach advanced cell divisions. A) The probability that a dividing precursor generates a daughter cell in division N was calculated under maximally stimulating conditions (2 µg/ml anti-CD3/28, 65 hours) and represented as a bar graph (n = 6 for all strains). Differences in response were most evident in later divisions (*p<0.05, B6 vs all other strains, ANOVA followed by Bonferroni post-test). B) Dose Responsiveness of autoimmune CD8 T cells to anti-CD3 mediated stimulation. The number of mitoses per 10,000 CD4 T cells was determined for cultures stimulated with a range (0–2 µg/ml) of anti-CD3 concentrations; each culture received 4 µg/ml anti-CD28. The percent of the maximal mitosis is shown plotted against the dose of anti-CD3. The NOD CD8 T cells exhibit a dose-response curve that diminishes more rapidly than the other strains. Significant differences in response are noted at the lower concentrations of anti-CD3 (n>6 for all strains, *p<0.01, NOD vs all other strains, ANOVA followed by Bonferroni post-test). Presented data are obtained from two separate experiments.

Having determined a common immune alteration in the CD4 compartment among the autoimmune strains, it remained to be seen whether discriminating immunologic criteria could be elucidated in the CD8 compartment. We applied the same analytical methods described above for the activation of CD4 cells. Both dose-response curves and probability profiles were generated following 65 hours of stimulation with anti-CD3 and anti-CD28 antibodies. As seen in Figure 6a, all three autoimmune strains demonstrate an ability to generate CD8 daughter cells in later generations of cell division. While this ability is slightly diminished compared to the non-autoimmune strain B6, it is not as marked as seen in the CD4 compartment (Fig. 6a). Intriguingly, the dose response curves serve to distinguish easily the NOD CD8 cells from their autoimmune counterparts (Fig. 6b). While both the MRL and NZBW strains exhibit a dose-response curve which overlays well with the normal strain, the NOD dose-responsiveness is markedly diminished. As was the case in the CD4 compartment, the NOD CD8 cells are about 4-fold less responsive than the normal strain. This finding has implications for the differentiation of the antigen activated peripheral CD8 cell compartment.

## Discussion

We have demonstrated a shared alteration in the peripheral immune system of three autoimmune prone murine strains. In our analysis, CD4 T cells exhibit proliferative arrest in early cell divisions and hence may not be subjected to the tolerance-inducing mechanisms that rely on multiple rounds of cell division [14], [15]. In addition, the diminished dose responsiveness of these CD4 T cells may further hinder their ability to become tolerant to autoantigens present at low density or poorly presented by the restricting MHC. The combination of these two deficiencies indicates a profound reduction in responsiveness to T cell stimuli. The dose response phenomenon may also be a critical functional parameter itself as the concentration at which autoantigens are present *in vivo* remains unknown. The distinction in the autoimmune dose response curves indicates how a similar antigen at a similar concentration may elicit distinct responses within different murine strains. Given the discrepancy in dose responsiveness even identical antigens may be perceived differently by these autoimmune prone immune systems and hence may initiate an inappropriate response in the periphery or fail to elicit the appropriate response at the thymic level. The latter interpretation is supported by the study of Lang and Bellgrau demonstrating CD4 hypoactivation in autoimmune prone mice and rats and correlation of this phenotype to altered thymic selection [32]. We have similarly previously reported the activation parameters of non-autoimmune BALB/c mice, which matched the B6 data presented here [16]. Previous studies also demonstrated that the NOD hypoproliferative phenotype was not restricted to anti-CD3, but could also be observed with superantigen SEA and in vivo to islet antigens [16], [33]. In addition, these data suggest that lowering the concentration of presented autoantigen may also profoundly limit T cell activation in strains predisposed to autoimmunity. In this regard, exogenous antigen or even infection may prevent autoimmunity by occupying MHC that would otherwise present self-antigen, a possibility that could alternatively explain the findings that constitute the “hygiene hypothesis”. Moreover, the difference in CD8 dose response, which is isolated to the NOD murine strain, may promote differences between the CD8 compartment response to self-antigens among autoimmune prone murine strains. This feature may explain how diabetes is driven along a pathway of cell mediated destruction of islets and other tissues while the two lupus-prone strains experience a disease course dominated by autoantibody production. It is also of interest that diabetes can be adoptively transferred by either CD4 or CD8 effector cell while murine lupus relies most heavily on the CD4 compartment [34], [35].

While the data indicated a profound diminishment in T cell responsiveness, the origin of this phenotype remained unclear as the stimulatory condition used, soluble mitogenic antibody, required the coordinated interplay of T cells and APCs. In particular, a number of studies have indicated that the B cell compartment within these strains is required for disease progression. Previous analysis in our laboratory suggested that T cells within the NOD mouse rely upon B cells for requisite co-stimulatory signals [16], [33]. We have now extended this finding to the lupus prone NZBWF1 and MRL strains. These data suggest that within these strains, the non-B APC compartment, which may include both macrophages and dendritic cells, are deficient in their ability to interact with the T lymphocyte compartment. These data also suggest that tolerance cannot be effectively maintained solely through interaction with antigen presented by the B lymphocyte compartment as has been suggested [36], [37], [38]. Rather it appears likely that the maintenance of a functional state of tolerance requires robust contribution from both B cells and other APCs. These data suggest that the critical contribution from professional APCs may be frequently missing in autoimmune prone murine strains. At the same time, the reliance upon B cells for co-stimulation and T cell activation may also represent an important opportunity. If B cells are required for promoting autoimmune disease, then targeted reprogramming of the B cell compartment may be sufficient to produce long-term disease amelioration. Such findings have been seen in diabetic NOD mice treated with CD20 depleting antibody [39]. Similarly, the induction of regulatory B cells has also shown promise in combating autoimmunity [40]. In patients with disease, the situation may be more complex and professional APC function may be more normal. Thus, B cell focused therapies may be relatively more able to combat autoimmunity in the murine models than in patients.

Some additional consideration for the expected *in vivo* activity may be gathered from considering a reported transgenic system in the setting of autoimmune diabetes. The islet reactive transgenic CD4 T cell specificity denoted BDC2.5 has previously been reported and initial characterization demonstrated the development of diabetes in mice expressing this receptor [41]. Backcrossing of this transgene fully onto NOD, where there is well known T-cell hyporesponsiveness, resulted in mice which paradoxically had diminished diabetes progression [42]. In contrast, B6 mice when bred to express both the selecting MHC (I-A^g7^) and the BDC2.5 transgene develop rapid diabetes. While indirect, these data would support the interpretation that T cell hyporesponsiveness may be a trait bred onto autoimmune animals to retard the development of autoimmune disease until after the reproductive period. Resolving the nature of the T cell response in the context of autoimmunity is of critical importance to the design of future clinical interventions. On the one hand, activating T cells may promote the development of tolerance while, on the other, it may exacerbate disease; a rational choice demands an understanding of the underlying T cell phenotype.

Overall, our data suggest a model in which tolerance may be broken initially at the CD4 level as a result of inappropriate integration of stimulation signals. The development of autoimmune effector mechanisms and differential progression to a diabetes or lupus phenotypes then depends on subsequent cellular interactions and can take advantage of derangements in other cellular compartments. B lymphocytes may serve as a critical intermediary in this process. Therapeutic modalities directed at improving CD4 activation may prevent the initial loss of tolerance; however, CD8 specific therapies may be particularly advantageous in obviating the cell mediated destruction that characterizes diabetes progression.

## Materials and Methods

### Ethics Statement

All experiments were conducted in compliance with the guidelines for the care and use of laboratory animals and approved by the IACUC at Vanderbilt University (protocol M/10/282).

### Mice

NOD/LtJ (H-2^g7^), NZBW(H-2^d/z^), MRL (H-2^k^), and C57BL/6 (H-2^b^) mice were purchased from the Jackson Laboratories (Bar Harbor, Maine). All mice were housed under specific pathogen-free barrier conditions and were analyzed at 8–11 weeks of age, prior to the onset of clinical disease symptoms.

### CFSE labeling of lymphocytes

Lymphocytes were labeled with 5-(and −6)-carboxyfluorescein diacetate succinimidyl ester (CFSE) (Molecular Probes, Eugene, OR) as previously described [43], [44]. Briefly, splenocytes were resuspended at a concentration of 10×10^6^ cells/ml in serum-free IMDM (Gibco/BRL, Gaithersberg, MD) at 37°C. An equal volume of a 1∶350 dilution of the CFSE stock (5 mM in DMSO) in 37°C serum-free IMDM was then added to the cell preparation, which was subsequently incubated for 5 minutes at 37°C. CFSE labeling was quenched by adding an equal volume of heat-inactivated FCS (HI-FCS), whereupon cells were washed twice and resuspended in IMDM containing 10% HI-FCS.

### In vitro T cell stimulations

CFSE-labeled cells were plated in 24 well plates at a density of 1×10^6^ total cells in 1 ml of Iscove’s modification of Dulbecco’s medium (IMDM) supplemented with l-glutamine, 2-ME, 10% HI-FCS, and the designated amount of anti-CD3 (145–2C11) and anti-CD28 (37.51) antibodies. All cells were incubated for 65–70 h at 37°C in 5% CO_2_. After incubation, the cultured cells were harvested and stained with allophycocyanin (APC) -conjugated anti-CD4 (RM4–5) to allow the identification of CFSE-labeled CD4+ T cells using FACSCalibur^®^ (Beckton Dickinson, Mountain View, CA). 10,000 CD4+ events were collected and division was tracked utilizing a live cell gate which included the blasting cells, as determined by forward and side scatter. Division history of CD4+ and CD8+ T cells was analyzed as previously described, based on the property of CFSE-labeled cells to lose half of their fluorescence intensity with each round of division [45]. Dead cells in the divided, CD4+ gate were discriminated by 7AAD positivity.

### Calculation of Probability Distribution and Mitotic Number

The data generated by CFSE labeled cultures was analyzed to determine the potential for activation inherent in the precursor pool. To perform this calculation, we determined the number of activated precursors required to produce all the daughter cells in a given division peak (P), a factor we term precursor equivalents, by using the following formula:


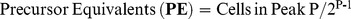
 where P is the Peak number.

The probability of generating a daughter cell in peak P is given by dividing the number of precursor equivalents for that division by the total number of equivalents for all divided peaks (P>1).

Mitosis was calculated using a standard formula as follows:

#Mitoses = ∑ (2 ^(p–1)^–1)(N_p_/2^(p–1)^) where the sum is completed over all peaks, p represents the peak number, and N_p_ represents the normalized number of cells in peak p.

### Plate Bound Stimulation Assay

Plate bound stimulation was performed with cells prepared as described above for polyclonal stimulation with soluble antibody. Twenty -four hours prior to the addition of cells, anti-CD3 and anti-CD28 antibodies at a concentration of 2 µg/ml were plated on 24 well culture plates in serum free IMDM. The plates were stored at 4°C throughout the binding process.
